# MRI-based radiomics to predict lipomatous soft tissue tumors malignancy: a pilot study

**DOI:** 10.1186/s40644-020-00354-7

**Published:** 2020-10-28

**Authors:** Benjamin Leporq, Amine Bouhamama, Frank Pilleul, Fabrice Lame, Catherine Bihane, Michael Sdika, Jean-Yves Blay, Olivier Beuf

**Affiliations:** 1 Univ Lyon, INSA-Lyon, Université Claude Bernard Lyon 1, UJM-Saint Etienne, CNRS, Inserm, CREATIS UMR 5220, Villeurbanne, France; 2 Department of Radiology, CRLCC Léon Berard, Lyon, France; 3 Department of Oncology, CRLCC Léon Berard, Lyon, France

**Keywords:** Magnetic resonance imaging, Radiomic, Liposarcoma

## Abstract

**Objectives:**

To develop and validate a MRI-based radiomic method to predict malignancies in lipomatous soft tissue tumors.

**Methods:**

This retrospective study searched in the database of our pathology department, data from patients with lipomatous soft tissue tumors, with histology and gadolinium-contrast enhanced T_1_w MR images, obtained from 56 centers with non-uniform protocols. For each tumor, 87 radiomic features were extracted by two independent observers to evaluate the inter-observer reproducibility. A reduction of learning base dimension was performed from reproducibility and relevancy criteria. A model was subsequently prototyped using a linear support vector machine to predict malignant lesions.

**Results:**

Eighty-one subjects with lipomatous soft tissue tumors including 40 lipomas and 41 atypical lipomatous tumors or well-differentiated liposarcomas with fat-suppressed T_1_w contrast enhanced MR images available were retrospectively enrolled. Based on a Pearson’s correlation coefficient threshold at 0.8, 55 out of 87 (63.2%) radiomic features were considered reproducible. Further introduction of relevancy finally selected 35 radiomic features to be integrated in the model. To predict malignant tumors, model diagnostic performances were as follow: AUROC = 0.96; sensitivity = 100%; specificity = 90%; positive predictive value = 90.9%; negative predictive value = 100% and overall accuracy = 95.0%.

**Conclusion:**

This work demonstrates that radiomics allows to predict malignancy in soft tissue lipomatous tumors with routinely used MR acquisition in clinical oncology. These encouraging results need to be further confirmed in an external validation population.

## Introduction

Lipomatous soft tissue tumors are rare tumors arising from benign (such as lipomas) or malignant (liposarcomas) mesenchymal tissue proliferation. Liposarcomas constitute the second most common subgroup of soft tissue sarcomas in adults, with a peak incidence among patients between the fifth and seventh decade (10–35%) [1, 2]. The World Health Organization defines four histologic liposarcoma subtypes: well differentiated (WDL), myxoid (including myxoid with round cell component), dedifferentiated, and pleomorphic liposarcoma [3]. Benign lipomas are treated with marginal resection or simple medical follow-up, borderline lesions such as well-differentiated liposarcomas and atypical lipomatous tumors (ALT) require a complete resection to prevent local recurrence and potential dedifferentiation [1, 4]. High-grade liposarcomas may require a multimodal therapy approach according to the aggressiveness of the tumor with large resection and concomitant chemotherapy and/or radiotherapy [1, 4]. Therefore, noninvasive diagnosis to differentiate benign lipomas from malignant lipomatous soft tissue tumors is crucial to guide the therapeutic strategy. Histology is the gold standard for the diagnosis of lipomatous soft tissue tumors. Nevertheless, specific imaging methods should be considered prior to conducting invasive procedures potentially risky, and preventing unnecessary patient exposure to expensive procedures. These ethical and clinical challenges related to biopsy-based assays can be addressed using medical imaging, routinely used for cancer diagnosis and staging in clinical oncology. However, tumor heterogeneity results in wide range of imaging appearance and reduces the performance of conventional imaging features to efficiently distinguish benign from malignant forms of lipomatous soft-tissue tumors. One of the greatest dilemma for a pathologist lies in differentiating lipoma from WDL or ALT. Brisson and colleagues reported a positive predictive value of 47% in the diagnosis of ALT/WDL using Magnetic Resonance Imaging (MRI) because of morphological overlap with many benign lipoma variants [5]. Therefore, many biopsies for benign lesions could be avoided and more specific methods are needed to enhance diagnosis.

In the past decade, the field of medical image analysis with an extensive amount of available data is evolving rapidly and resulted in the emergence of radiomics, a discipline deriving imaging features into mineable data (the radiome) for decision support. Radiomics aims to uncover tumor characteristics from different microscopic (genetic, molecular, cellular, histologic) to macroscopic scale [6–8]. The necessary condition is that the images used for radiome extraction (the radiomic fingerprint) translate the underlying pathological mechanisms. We aim to develop and validate a MRI-based radiomic method to specifically distinguish benign lipomas from malignant ALT or WDL.

## Patients and method

### Patients

This retrospective study searched the database of the Pathology department at our comprehensive cancer center to identify patients with lipomatous soft tissue tumors, with histology and gadolinium-contrast enhanced T_1_w MR images available. The study was approved by our institutional review board and the requirement to obtain informed consent was waived.

### MRI data

MR images were collected from 56 different centers with non-uniform protocols and centralized in the Picture Archiving and Communication System (PACS) of our cancer center. Acquisition were performed at three different fields (1.0 T, 1.5 T, and 3.0 T) with 18 MR systems commercialized by four vendors (*Siemens Healthineers, Erlangen, Germany*, 39.5%; General Electric Healthcare, *Milwaukee, WI, USA*, 33.3%; *Philips Healthcare, Best, The Netherlands*, 24.6%; and *Toshiba medical systems corporation, Otawara, Tochigi, Japan*, 2.6%). Image acquisitions were mainly acquired using a 2D fast spin-echo sequence (53.3% with a fat saturation and 11.7% with a fat-water decomposition) (65%), a 3D isotropic fast spin-echo sequence (4.9%), a 3D gradient echo sequence (16.5% with a fat saturation and 13.6% with a fat-water decomposition) and in 35% of cases with. Mean pixel size was 0.81^2^ ± 0.29^2^ mm^2^ (0.37^2^–1.75^2^).

### Images processing

Images were automatically loaded in in-house software developed on Matlab R2017a (The MathWorks, Natick, USA). To evaluate the inter-observer reproducibility, the tumor was manually segmented by two independent observers blinded to histology: an experimented physicist (BL, 13-year experience in MR imaging) and an experimented radiographer (CB, 19-year experience in MR imaging). Tumor segmentation in two dimensions were performed. The slice level with the longest tumor diameter was selected.

**Fig. 1:**
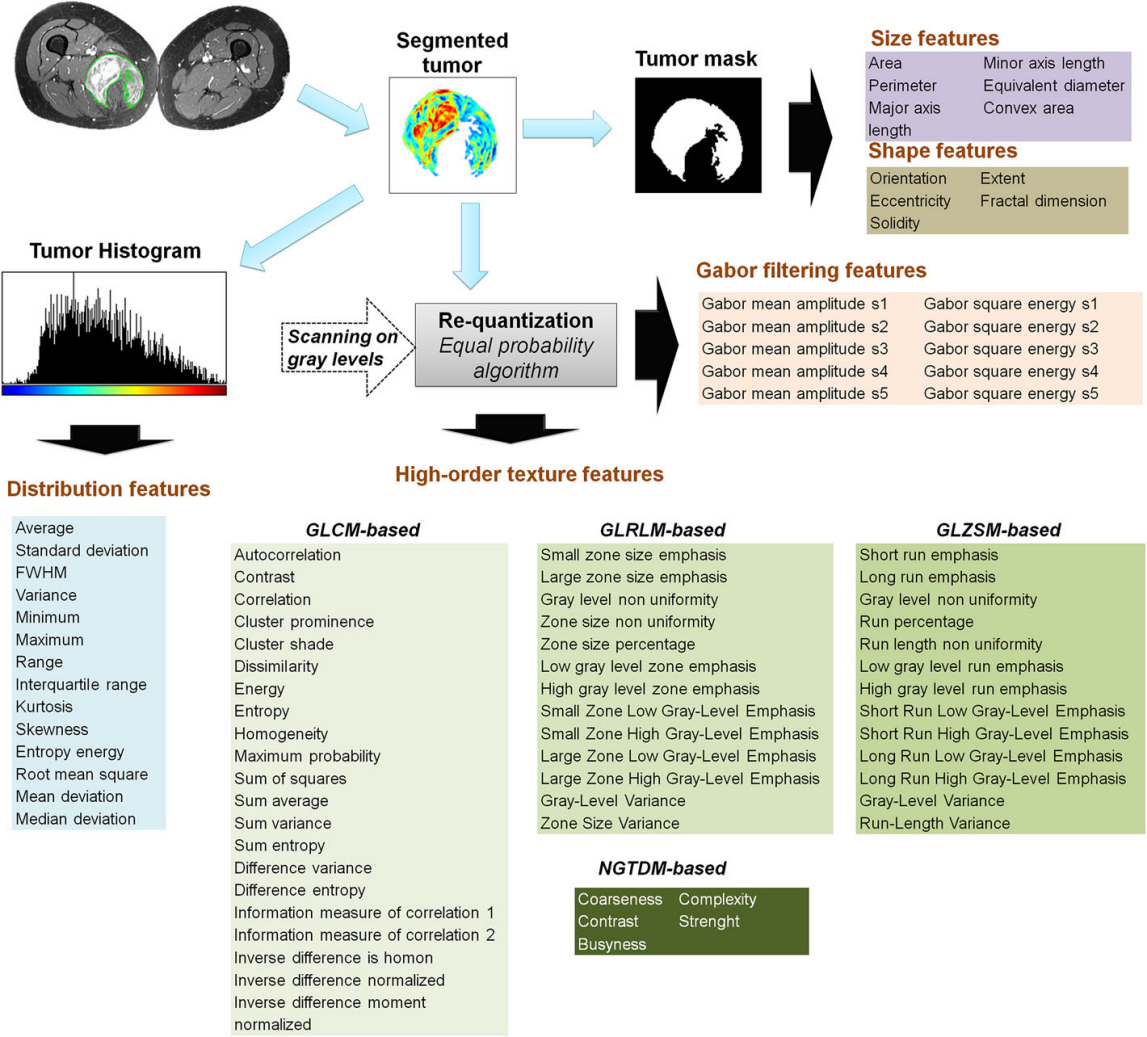
Radiome extraction pipeline. Size and shape features were extracted from the binary mask. Intensity distribution features were extracted from masked MR images from the histogram built with 256 bins. Image gray levels were discretized in a smaller number of gray levels with an equal probability algorithm. Images were discretized in 8, 16, 24, 32, 40, 48, and 64 Gy levels. For each discretization level, four matrices were built: GLCM (Gray-level co-occurrence matrix), GLRLM (Gray-level run length matrix), GLSZM (Gray-level size zone matrix), and NGTDM (Neighborhood gray tone difference matrix) from which characteristics were extracted, then averaged. Frequency domain-based texture features were extracted using a Gabor filtering

Tumor mask was next applied on fat-suppressed enhanced MR images and 87 radiomic features were extracted. They included size, shape, intensity distribution, image domain, and frequency domain textures features. The full list of features is summarized in Fig.1.

Size and shape features were directly extracted from the binary mask. Intensity distribution features were extracted from masked MR images without normalization or filtering of voxel intensities, and from the histogram built with 256 bins. Before the extraction of texture features, images gray levels were discretized in reduced number of gray levels. This operation was done using an equal probability algorithm to define decision thresholds for the volume in order to reach the same number of voxels for a given reconstructed level in the quantized volume, for all gray levels. Images were discretized in 8, 16, 24, 32, 40, 48 and 64 Gy levels and for each discretization level four matrices were built: Gray-level co-occurrence matrix (GLCM), Gray-level run length matrix (GLRLM), Gray-level size zone matrix (GLSZM) and Neighborhood Gray Tone Difference Matrix ((NGTDM) for characteristics extraction (Table.1) as previously reported [9–15]. Frequency domain-based texture features were extracted using a Gabor filtering. GLCM and GLRLM were computed for 4 directions (0°, 45°, 90°, and 135°) with an offset of 1 pixel. For GLSZM and NGTDM, a 26 pixel connectivity was used. For Gabor filtering, 5 scales, 6 orientations, and a minimal wavelength of 3 were used (Fig.1).

**Table 1:** Linear regression parameters (Pearson correlation coefficient (r), coefficient of determination (R^2^) and Spearman’s rank-order coefficient (*ρ*)) computed to evaluate the inter-observer variability according to the radiomics features family

	Size	Shape	Intensity distribution	GLCM	GLRLM	GLZSM	NGTDM	Gabor filtering
**r**	0.89 ± 0.04 (0.84–0.93)	0.58 ± 0.20 (0.27–0.89)	0.90 ± 0.16 (0.49–0.99)	0.77 ± 0.08 (0.59–0.86)	0.76 ± 0.13 (0.57–0.95)	0.77 ± 0.17 (0.49–0.99)	0.82 ± 0.08 (0.73–0.93)	0.98 ± 0.02 (0.96–0.997)
**R**^**2**^	0.80 ± 0.08 (0.70–0.86)	0.38 ± 0.24 (0.07–0.79)	0.83 ± 0.24 (0.24–0.98)	0.6 ± 0.12 (0.35–0.74)	0.6 ± 0.2 (0.33–0.89)	0.62 ± 0.26 (0.24–0.99)	0.67 ± 0.13 (0.54–0.86)	0.96 ± 0.04 (0.92–0.99)
***ρ***	0.92 ± 0.01 (0.90–0.93)	0.62 ± 0.17 (0.46–0.93)	0.86 ± 0.06 (0.72–0.92)	0.77 ± 0.04 (0.68–0.81)	0.74 ± 0.06 (0.57–0.81)	0.74 ± 0.01 (0.53–0.84)	0.87 ± 0.06 (0.77–0.92)	0.88 ± 0.02 (0.85–0.91)

Values reported are mean ± standard deviation (ranges)

### Evaluation of inter-observer variability and effect of gray level discretization

The inter-observer reproducibility was analyzed using regression models. Pearson’s correlation coefficient (r), coefficient of determination (R^2^), and Spearman’s rank-order correlation coefficient (*ρ*) were computed.

The effect of the number of gray levels for image discretization was studied by computing the coefficient of variation (CV) for each image domain texture features from values issued from each gray level discretization (i.e. 8, 16, 24, 32, 48, and 64) in 10 subjects per group randomly chosen. Next, feature-by-feature, the average CV on all tested subjects was determined.CV-values from 0 to 5% were assimilated as an absence of variation, and CV-values from 5 to 25% were considered as an acceptable variation. CV-values higher than 25% indicated unacceptable variations.

### Prediction of malignant lipomatous tumors

To predict malignant tumors, a classification model was built using supervised machine learning. The first step reduced the initial set of features to minimize the problem of overfitting and created another set of relevant features in term of relevancy and inter-observer reproducibility through filtering with a double thresholding on t-test *p*-value and Pearson’s correlation coefficient, previously computed through reproducibility study. Threshold-values were p-value smaller than 0.2, and Pearson’s correlation coefficient higher than 0.8. The threshold for *p*-value was chosen as a compromise between relevancy and stringency faced with dimensionality reduction to be consistent with observations size.

The second step performed a prototyping of classification model with a support vector machine as a classifier with a linear kernel. Before training, data were centered at their mean and scaled to have unit standard deviation. Support vector computation and hyperplane separation were done using a sequential minimal optimization. The classes were well balanced; a unit box constraint was used for soft margin. To evaluate the overfitting, cross validation used the holdout method (75% of data were used for training set and 25% for test set). Similar ratio between benign and malignant lesions was used in both dataset (around 50 vs. 50% in training and test set).

### Effect of MR scanner vendor on features

The effect of MR vendors on the set of texture features integrated in the model was investigated. For each group, features were stratified by MR vendors and compared using the one-way ANOVA approach with Bonferroni’s multiple post-test comparisons.

## Results

### Patients

A total of 81 patients with lipomatous soft tissue tumors were enrolled in this study, 40 lipomas and 41 -differentiated liposarcomas and atypical lipomatous tumors.

### Inter-observer reproducibility and effect of number of gray level discretization

For the whole set of radiomic features, the average Pearson’s correlation coefficient was 0.81 ± 0.15 [0.27–0.99]; Determination and Spearman ρ average coefficients were 0.68 ± 0.23 [0.07–0.99], and 0.79 ± 0.10 [0.46–0.93], respectively. Based on a threshold at 0.8 for the Pearson’s correlation coefficient, 55 (63.2%) of 87 radiomic features were considered reproducible. Stratified by feature family, shape features were the least reproducible (r = 0.58 ± 0.20; R^2^ = 0.38 ± 0.24; *ρ* = 0.62 ± 0.17; 17% of features considered reproducible). Gabor filtering features were the most reproducible (r = 0.98 ± 0.02; R^2^ = 0.96 ± 0.04; *ρ* =0.88 ± 0.02; 100% of features considered reproducible). Results for all features families are summarized in Table 1.

Based on CV-values, the number of gray level impacted 40 (76.9%) of 52 images domain based texture features. Twelve (23.1%) out of 52 features had acceptable variation including 5 out of 12 features (41.6%) with a CV smaller than 5%, considered as totally not impacted by gray level discretization. These features were the following: “Correlation”, “Information measure of correlation”, “Inverse difference normalized”, “Inverse difference moment normalized”, and “Coarseness”. By texture family, the second order texture features (GLCM -based) were the least impacted (CV =59.8 ± 50.6% [0.37–154%]). High order texture features were more impacted: CV =69.0 ± 38.2% [10.8–132%]; CV =95.6 ± 58.6% [18.5–209%]; CV =89.1 ± 61.1% [4.72–158%] for GLRLM, GLSZM and NGTDM texture features respectively.

### Prediction of malignant lipomatous tumors

**Fig. 2:**
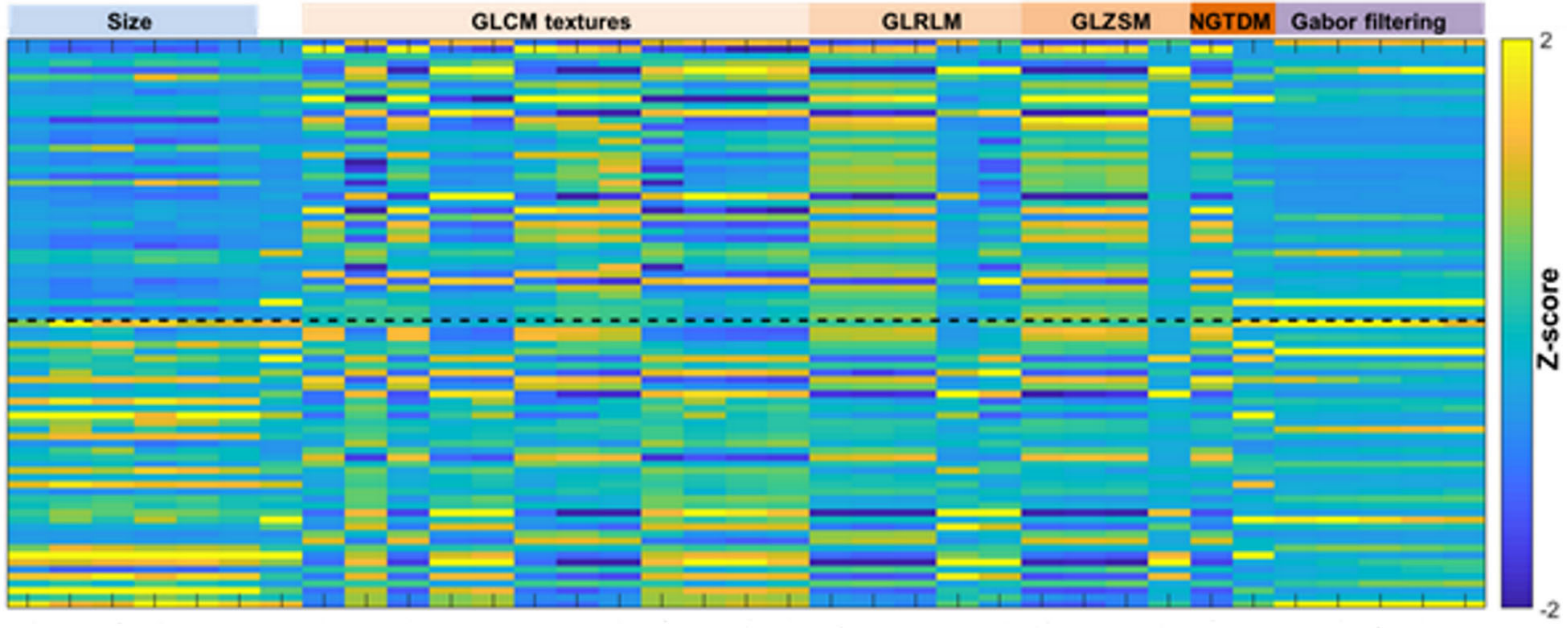
Heatmap representing the reduced learning base after features filtering from reproducibility and relevancy criterion. From the initial feature set, only 35 features were integrated. Size and high order texture features were largely integrated whereas shape and intensity distribution features were not integrated due to poor reproducibility and relevancy, respectively. Black dash line represents the limit between the two classes

**Fig. 3:**
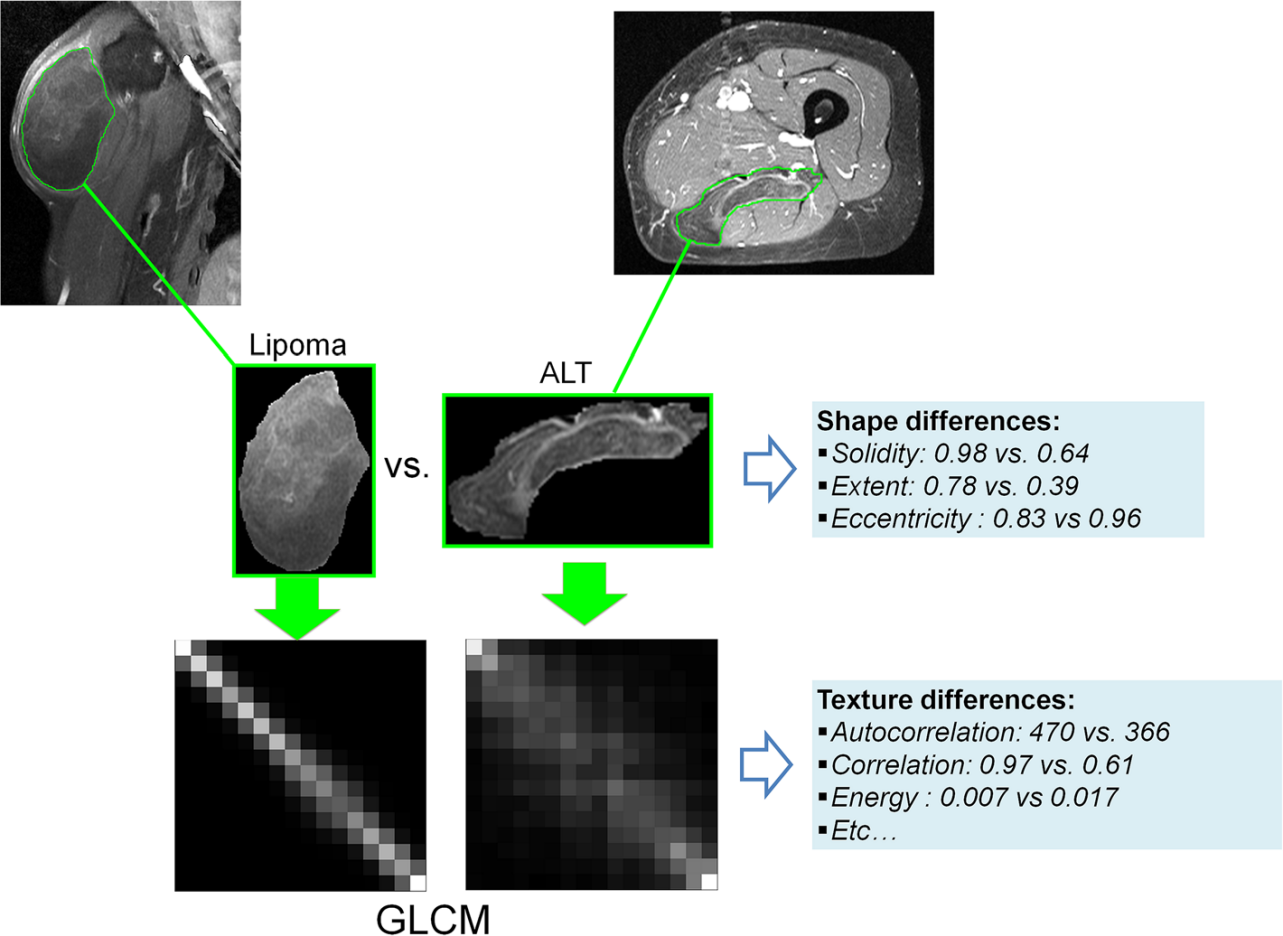
Malignant ALT (Atypical Lipomatous Tumors) display visual differences in shape comparison with lipoma. These differences were quantified by shape radiomics features (such as solidity, extent, and eccentricity), and expressed in the radiome. Tumor enhancements display different texture which can be recorded by the GLCM (Gray-level co-occurrence matrix); quantified using GLCM-based descriptors and expressed in the radiome

Based on t-test *p*-value, out of 87 features, 64 (73.6%) were included in the reduced feature set. Size features were the most relevant (100% of relevant features), intensity distribution were the worst relevant (28.6% of relevant features). Results for each family are summarized in Table 2. After combination with reproducibility criterion, the radiome was finally reduced to 35 (40.2%) features. Details on preselected features were provided in Fig. 2. An example illustrating radiomic features variations according to benign or malignant status is provided in Fig. 3. To distinguish benign from malignant tumors, model diagnostic performances were as follow: AUROC =0.96; sensitivity =100% (95%CI 100–100%); specificity =90% (95%CI 71.4–108%); positive predictive value =90.9% (95%CI 73.9–108%); negative predictive value =100% (95%CI 100–100%), and overall accuracy =95.0% (95%CI 85.5–105%).

**Table 2:** Percentage of relevant features to discriminate benign from malignant lipomatous tumors

	Benign vs. Malignant
**Size**	100% (6/6)
**Shape**	80.0% (4/5)
**Intensity distribution**	28.6% (4/14)
**GLCM**	95.2% (20/21)
**GLRLM**	92.3% (12/13)
**GLSZM**	69.2% (9/13)
**NGTDM**	80.0% (4/5)
**Gabor filtering**	50.0% (5/10)

A t test *p* < 0.2 was considered for relevancy

### Effect of MR scanner vendor on features

The benign group showed no significant differences according to the different manufacturers. In the malignant group, 3 GLCM texture-based features displayed significant differences (“Contrast”, “Dissimilarity”, and “Difference variance”). These differences were systematically identified between General Electric and Philips MR systems. No significant differences in pixel sizes were observed according to the different MR vendors and between the groups, regardless of MR vendors.

## Discussion

This work showed that routine MRI acquisition allows to evaluate lipomatous soft-tissue malignancy in clinical practice with promising diagnostic performances. Our radiomic analysis used fat-suppressed contrast enhanced T_1_-weighted images collected from 56 institutions. Our aim was to differentiate lipomas from WDL/ALT, considered as the most challenging issue in clinical practice. To our knowledge, this study is the first to report MR-based radiomic results to evaluate lipomatous tumor malignancy. According to these results, all lesions were detected with 100% sensitivity and a good specificity (90%) suggesting that radiomics may help to decrease the number of unnecessary biopsy. This issue is central for ethical and healthcare cost-effective purpose.

The gadolinium uptake used a single radiomic fingerprint. The radiome only gathered information translating vascular and necrosis processes. Oedema and fat content have also been described to discriminate lipomas from liposarcomas [9, 16–19]. Therefore, the combined use of suppressed T_2_-weighted MR imaging and/or no fat-suppressed acquisition could still improve diagnostic performances reported in the present study. However, such MRI acquisitions are not systematically required in clinical protocols.

Size features were found relevant to predict malignant lipomatous tumors and were largely integrated in the models. This illustrated that tumor size may help to characterize the malignancy as reported in previous works based on conventional imaging characteristic [16, 18–24]. Texture features (particularly GLCM-based) were largely reported as relevant features, whereas intensity distribution features were not strongly appropriate, and demonstrate that the spatial heterogeneity of gadolinium enhancement is more discriminant than its intensity to isolate benign lesion. This could be explained by the presence of a structured capillary network and/or nodular septa in malignant lesions. This is also consistent with clinical practice based on visual characterization of tumor enhancement homogeneity and the presence of thick and nodular septa as key characteristics [5, 19]. We reported poor inter-observer reproducibility, shape features were consequently not integrated, despite a good relevancy. Shape differences observed here may also reflect excessive cell proliferation in malignant tumors [25]. Tumor shape belong to key characteristics visually assessed in clinical practice [18, 19].

The implementation of multivariate scores issued from the combination of conventional MR imaging features to distinguish benign lipomas from ALT alone or ALT/WDL have been previously reported [19, 20]. However, features were subjective and only qualitative. These scores may be strongly exposed to inter-observer variability and may depend on the experience of the radiologist. However, such previous results clearly demonstrated the interest of combining different quantitative features expressing subjective characteristics, such as radiomics does.

Our results suggested that radiomic features are also exposed to inter-observer variability. The segmentation step was strongly assumed to introduce these discrepancies as the single non-automated step in the processing pipeline. Based on the Pearson’s correlation coefficient, only 63% of features were found to be reproducible. Features were not equally impacted. Shape features were the most impacted, which may be explained by an observer disagreement in the choice of slice level for tumor contouring. Size and Gabor filtering texture features were less impacted with 100% of features considered as reproducible. To prospectively classify lesion groups, the inherent observer variability in the data mining step should be carefully considered. We subsequently proposed to discard features from the initial radiome by thresholding on Pearson’s correlation coefficient and implement the model from a reduced data set. We have also investigated the effect of the number of gray level for image discretization on texture features stability and showed a strong impact of discretization with 77% of features impacted. GLCM based-texture seem to be less impacted than higher order features based on GLRLM, GLZSM and NGTDM features, as previously reported [26, 27]. This result suggested that method standardization for feature extraction is mandatory for prospective use in multicentric trials or for retrospective use of annotated data set. In parallel, selection of number of gray level impacted the feature relevancy for classifying. However, this hypothesis was not explored neither evaluated in this study. Therefore, we have proposed to introduce the number of gray level used for the discretization in a second dimension by quantifying texture features on a discrete range of gray level (between 8 to 64), and used the average as final values for each texture features. Out of the integrated features, a slight vendor effect was observed, mainly between General electric and Philips MR systems and limited for GLCM texture-based features. Other factors, regardless MR vendors could introduce pixel value differences such as the repetition time, the injected dose of gadolinium, the delay between injection and acquisition, and the fat saturation procedure. These outcomes were not directly investigated, but the model prototyped from these heterogeneous data has provided indirect evidence on these issues.

This study has several limitations. First, image resolution has also been reported to impact features stability and we did not use a resampling procedure to normalize pixel size in this work. However, pixel size displayed no significant differences between groups.

Second, although internal cross-validation on test and application data was used to build the model, no external validation on independent population was performed. In this pilot study, the multicenter multi-vendor aspect in the database for subsequently integration of the maximum of acquisition heterogeneities in our model has been preferred. As a consequence, these encouraging results need to be further confirmed on multicenter prospective or applied to existing population to rigorously verify the impact of MR-vendor and MR protocol on classification performances.

Third, segmentation was performed only in two dimensions. The rationale supporting two-dimension segmentation rather than a three-dimension was to simplify the annotation step. This choice may have enhanced feature inter-observer variability, particularly for shape features. In addition, an inherent risk of sampling effect may have been subsequently generated. These issues need to be further investigated.

## Conclusion

To conclude, this work demonstrates that radiomics allows to predict malignancy in soft tissue lipomatous tumors with routine MR acquisition in clinical oncology. These encouraging results need to be further confirmed in a validation population.

## Abbreviations

WDL: Well-Differentiated Lipomas
ALT: Atypical Lipomatous Tumors
MRI: Magnetic Resonance Imaging
GLCM: Gray-Level Co-occurrence Matrix)
GLRLM: Gray-Level Run Length Matrix),
GLSZM: Gray-Level Size Zone Matrix)
NGTDM: Neighborhood Gray Tone Difference Matrix)
CV: Coefficient of Variation
AUROC: Area Under the Receiver Operating Curve
